# Supplementary material for: Three-year outcomes of the randomized phase III SEIPLUS trial of extensive intraoperative peritoneal lavage for locally advanced gastric cancer
Source: Nat Commun. 2021 Nov 15;12:6598. doi: 10.1038/s41467-021-26778-8 (PMC8594430; doi:10.1038/s41467-021-26778-8)
Supplement: Supplementary file 1 — Supplementary Information [file 41467_2021_26778_MOESM1_ESM.pdf]

Supplementary Note 1
Study Protocol

Combining surgery and extensive intraoperative peritoneal lavage versus surgery alone for locally advanced gastric cancer (SEIPLUS): A randomized controlled trial

Principal Investigator:

Dazhi Xu, PhD, MD, Fudan University Shanghai Cancer Center

Version 2.0

Date: 2020-10-01

TABLE OF CONTENTS

Study Team Roster 3

Participating Study Centers 5

Study Protocol 6

- Study Title 6
- Study Status 6
  - Study Start 6
  - Study Completion 6
- Background 6
- Objectives 8
- Outcomes 8
  - Primary End Point 8
  - Secondary End Points 9
- Statistical Considerations 9
- Arms and Interventions 9
- Selection and Enrollment of Participants 10
  - Inclusion Criteria 10
  - Exclusion Criteria 11

Participant Rights and Confidentiality 12

- Institutional Review Board (IRB) Review 12
- Consent Forms 12
- Participant Confidentiality 13

Quality Assurance 13

- Quality Control 13
- Ethics Committee 14
- Training 14

Reference 15

Study Team Roster

Dazhi Xu, PhD, MD (Principal Investigator), Fudan University Shanghai Cancer Center, Shanghai, China；Sun Yat-sen University Cancer Center, Guangzhou, Guangdong, China

Contact: xudzh@shca.org.cn

Jing Guo， MD, (Co-Investigator), Fudan University Shanghai Cancer Center, Shanghai, China； Sun Yat-sen University Cancer Center, Guangzhou, Guangdong, China

Xiaowei Sun, PhD, MD (Co-Investigator), Sun Yat-sen University Cancer Center, Guangzhou, Guangdong, China

Aman Xu, MD, (Co-Investigator), The First Affiliated Hospital of Anhui Medical University, Hefei, Anhui, China

Xuhui Zhao, MD, (Co-Investigator) Department of General Surgery, The First Affiliated Hospital of University of Science and Technology of China, Anhui Provincial Cancer Hospital, Hefei, Anhui, China

Yaming Zhang, MD, (Co-Investigator) Anqing Municipal Hospital, Anhui, China

Li Chen, MD, (Co-Investigator), The Second Affiliated Hospital of Zhejiang University School of Medicine, Zhejiang, China

Tao Zhang, MD, (Co-Investigator), Yuebei People's Hospital, Shaoguan, Guangdong, China

Gang Li, MD, (Co-Investigator), Jiangsu Cancer Hospital, Nanjing, Jiangsu, China

Huamin Rao, MD, (Co-Investigator), Jiangxi Provincial Cancer Hospital, Nanchang, Jiangxi, China

Rupeng Zhang，MD, (Co-Investigator), Tianjin Medical University Cancer Institute and Hospital, Tianjin, Tianjin, China

Hongtao Xu，MD, (Co-Investigator), Lishui Municipal Central Hospital, Lishui, Zhejiang, China

Participating Study Centers

1. Sun Yat-sen University Cancer Center
2. The First Affiliated Hospital of Anhui Medical University
3. The First Affiliated Hospital of University of Science and Technology of China
4. The First Affiliated Hospital of Wannan Medical College
5. Jiangxi Provincial Cancer Hospital
6. Anqing Municipal Hospital
7. Tianjin Medical University Cancer Institute and Hospital
8. The Second Affiliated Hospital of Zhejiang University School of Medicine
9. Yuebei People's Hospital
10. Jiangsu Cancer Hospital
11. Lishui Municipal Central Hospital

Study Protocol

Study Title

Combining surgery and extensive intraoperative peritoneal lavage versus surgery alone for locally advanced gastric cancer (SEIPLUS): A randomized controlled trial

Study Status

Study Start: March 2016 (At the end of March 2016, we completed the ethical review of each sub-center, held a kick-off meeting, and started online registration of SEIPLUS trial. The first patient was recruited on April 5, 2016.)

Study Completion: November 2020

Background

Gastric cancer is one of the most common cancers and remains the third leading cause of death among malignant tumors all over the world. Surgery is considered as the most effective treatment. While significant surgical technique and perioperative management have dramatically improved the survival of patients with advanced gastric cancer, patients

with T4 stage or serosal-positive gastric cancer often suffer from peritoneal recurrence, and the prognosis of those patients is poor. Peritoneal metastasis is caused by direct cancer cell dissemination from serosa-invasive tumors. Consequently, it is important to prevent peritoneal metastasis before the fixation of free cancer cells on the peritoneum.

In 2009, Kuramoto at el first reported that EIPL plus intraperitoneal chemotherapy could reduce PM significantly in AGC patients. The trial was intended to demonstrate the superiority in overall survival of addition of EIPL to standard treatment in patients with ≥T3 gastric cancer. Based on the 'limiting dilution theory', the peritoneal cavity is extensively washed 10 times with 1 L physiological saline at a time, followed by complete aspiration of the fluid. In this study, the EIPL-IPC group had a significantly lower incidence of peritoneal recurrence. Furthermore, the 5-year overall survival rate of the patients in the EIPL-IPC group (43.8%) was significantly better than that of the IPC group (4.6%) and the surgery-alone group (0%). Therefore, EIPL is easy, safe and inexpensive treatment. Gastrectomy with EIPL might be a new prophylactic strategy of peritoneal metastasis.

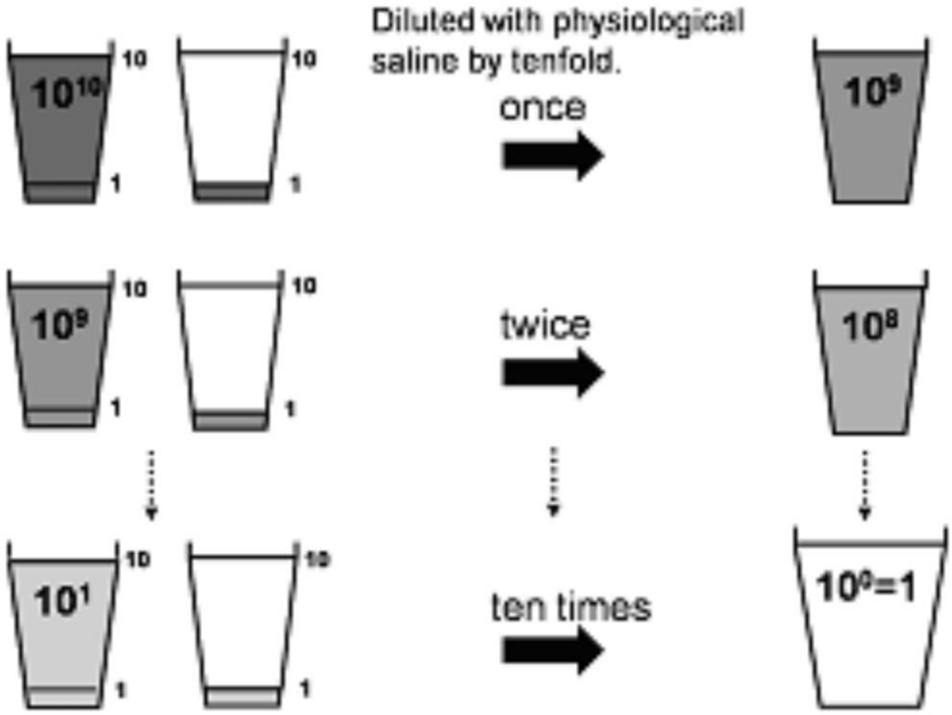

Objectives

Our study aims to explore the potential value of EIPL in improving the overall survival and disease-free survival for locally advanced gastric cancer after curative resection.

Outcomes

Primary End Point

3-year overall survival

Secondary End Points

3-year disease free survival, 3-year peritoneal recurrence-free survival，30-day postoperative complications and mortality.

Statistical Considerations

We calculated that 254 patients were needed in each group in order to detect a difference in 3-year overall survival of 60% for surgery alone group and 71% for EIPL group, with the use of the log-rank test, a two-sided alpha level of 0.05 and a power of 80%.

The differences between groups were compared by using chi-square tests and t test. All P values calculated in the analysis were two-sided. P values less than 0.05 were considered as statistically significant. Statistical analysis was performed using SPSS software, version 17.0 (IBM Corporation, Armonk, NY, USA).

Arms and Interventions

Experimental:

Extensive Intraoperative Peritoneal Lavage (EIPL), The peritoneal cavity of subject will be washed with 10 liters of warmed normal saline (1 liter per cycle for 10cycles). Every time, the peritoneal cavity was stirred

and washed sufficiently, and the fluid was aspirated completely.The abdomen will be closed as per standard.

No Intervention:

Standard Treatment (surgery alone), The peritoneal cavity of subjects will be washed with 3 liters or less of warmed normal saline. The abdomen will be closed as per standard.

All patients were recommended to undergo eight 3-week cycles of oral S-1 (40 mg/m² twice daily on days 1–14 of each cycle) plus intravenous oxaliplatin (100 mg/m² on day 1 of each cycle) postoperatively. Dose reductions or interruptions were used to manage potentially serious or life-threatening adverse events.

Selection and Enrollment of Participants

Inclusion Criteria

Preoperative Inclusion Criteria

1.Age older than 18 and younger than 80 years

2.ECOG PS 0 or 1

3.Written informed consent

4.Open surgery

5.cT3/4NxM0 at preoperative evaluation according to AJCC Cancer Staging Manual, 7th Edition

Intraoperative inclusion criteria:

1.cT3/4NxM0 according to the macroscopic appearance of exploratory laparotomy

2.R0 surgery

Exclusion Criteria

1. Previous neoadjuvant chemotherapy or radiotherapy
2. Positive peritoneal cytology
3. Peritoneal dissemination, distant lymph nodes, ovary, liver, lung, brain, and bone metastases
4. Massive ascites or cachexia
5. Participating in any other clinical trials currently
6. Severe cardiovascular, respiratory, kidney, liver and mental disease and diabetes
7. Poor compliance for adjuvant chemotherapy

Follow-up

All patients were followed up regularly and the follow-up data were registered. During the first 3 year of surgery, all patients were followed up by medical history, physical examination and blood testing with tumor markers every 3 months for the first 2 years and subsequently every 6 months. Chest and abdominal CT was performed every 6 months. Upper gastrointestinal endoscopy was performed annually. Recurrence was diagnosed by medical history and physical examination combined with imaging, cytology, or histology (if clinically needed).

Participant Rights and Confidentiality

Institutional Review Board (IRB) Review

The study protocol and the informed consent document for all clinic sites will be reviewed and approved by the Cancer Center of Sun-Yat Sen University. Individual site protocols will also be submitted for review and approval by the site local IRBs.

Consent Forms

Consent forms will be obtained from each participating provider. The consent form will describe the purpose of the study, the procedures to be

followed, the risks and benefits of participation, and compensation for participation.

Participant Confidentiality

Data will be recorded with the Crybyter protected web sites (https://crabyter.sinyoo.net/) to a data warehouse and transferred over secure network protocol. Only study investigators will have access to a list of study ID codes that will be traceable back to actual subject contact identifiers for clinicians.

Quality Assurance

Quality Control

Sponsor shall appoint monitors to conduct systematic monitoring for the study in accordance with Good Clinical Practice (GCP) principles to ensure the study is carried out according to the protocol and the case report form is identical with original data. The monitors should also assess compliance corresponding to the regulations and protocol. Investigators must ensure the integrity of the medical files. All study files will be reviewed by the sponsor and monitors. Monitors should ensure the

following: the rights of the subjects should be protected; original data should comply with GCP and protocol requirements.

Ethics Committee

Before study initiation, the study director must submit the study protocol, informed consent form and other related study documents to the Ethics Committees of every participating center to obtain its approval for conducting the clinical trial. After receiving the application, the Ethics Committee will convene a meeting to review, discuss and issue written comments attached with the list of participants, professional information and signature of primary investigator. During the study, the sponsor or contract research organization appointed by the sponsor should promptly report serious adverse events, including risks to subjects and other issues. Any modification to the protocol should be approved and recorded by the Medical Ethics Committee.

Training

Sponsor must ensure that all staff involved in the study have been trained by the sponsor or the organization designated by sponsor before the

study starts. An investigators' meeting should be convened for all investigators to be familiar with the protocol as well.

In the present study, all hospitals perform >100 D2 gastrectomy per year, and surgeons have sufficient experience for the surgery. Before the start of the study, standard operating procedures were predefined and given to all surgeons as text and figures. During the study, we made some SOP (standard operating procedure) cards for all centers to ensure the quality of procedures.

Reference

1. Mezhir JJ, Posner MC, Roggin KK. Prospective clinical trial of diagnostic peritoneal lavage to detect positive peritoneal cytology in patients with gastric cancer. J Surg Oncol.2013， 107(8):794-8.

2. Tang B, Peng ZH, Yu PW, et al. Aberrant expression of Cx43 is associated with the peritoneal metastasis of gastric cancer and Cx43-mediated gap junction enhances gastric cancer cell diapedesis from peritoneal mesothelium. PLOS One. 2013,8(9):e74527.

3. Hamazoe R1, Maeta M, Kaibara N. Intraperitoneal chemotherapy for prevention of peritoneal recurrence of gastric cancer. Final results of a randomized controlled study. Cancer.1994,73(8):2048-52.

4. Xu DZ, Zhan YQ, Sun XW, et al. Meta-analysis of intraperitoneal chemotherapy for gastric cancer. World J Gastroenterol. 2004,10(18):2727-30.

5. Sautner T1, Hofbauer F, Depisch D, Adjuvant intraperitoneal cisplatin chemotherapy does not improve long-term survival after surgery for advanced gastric cancer. J Clin Oncol.1994,12(5):970-4

6. Rosen HR1, Jatzko G, Repse S, et al. Adjuvant intraperitoneal chemotherapy with carbon-adsorbed mitomycin in patients with gastric cancer: results of a randomized multicenter trial of the Austrian Working Group for Surgical Oncology. J Clin Oncol.1998,16(8):2733-8；

7. Coccolini F1, Cotte E2, Glehen O2, et al. Intraperitoneal chemotherapy in advanced gastric cancer. Meta-analysis of randomized trials. Eur J Surg Oncol. 2014,40(1):12-26.

8. Kuramoto M1, Shimada S, Ikeshima S, et al. Extensive intraoperative peritoneal lavage as a standard prophylactic strategy for peritoneal recurrence in patients with gastric carcinoma. Ann Surg.2009, 250(2):242-6.

9. Misawa K1, Mochizuki Y, Ohashi N, et al. A randomized phase III trial exploring the prognostic value of extensive intraoperative peritoneal lavage in addition to standard treatment for resectable advanced gastric cancer: CCOG 1102 study. Jpn J Clin Oncol. 2014, 44(1):101-3.

10. Misawa K, Mochizuki Y, Ohashi N, et al. A randomized phase III trial exploring the prognostic value of extensive intraoperative peritoneal lavage in addition to standard treatment for resectable advanced gastric cancer: CCOG 1102 study. Jpn J Clin Oncol 2014; 44(1): 101-3.

11. Kim G, Chen E, Tay AY, et al. Extensive peritoneal lavage after curative gastrectomy for gastric cancer (EXPEL): study protocol of an international multicentre randomised controlled trial. Jpn J Clin Oncol 2017; 47(2): 179-84.

Supplementary Table 1. Univariate and Multivariable Cox Regression Analyses of Risk Factors for Survival

Variables	Overall survival				Disease-free survival			
	Univariate		Multivariable		Univariate		Multivariable	
	HR (95%CI)	p value	HR (95%CI)	p value	HR (95%CI)	p value	HR (95%CI)	p value
Procedure (Surgery alone vs Surgery+EIPL)	0.97 (0.76-1.25)	0.83	0.95 (0.74-1.22)	0.69	0.88 (0.70-1.11)	0.29	0.85 (0.67-1.07)	0.16
Age (≤60 vs >60)	1.67 (1.28-2.17)	0.0001	1.83 (1.40-2.39)	<0.0001	1.48 (1.16-1.89)	0.001	1.61 (1.26-2.05)	0.0002
Sex (Male vs Female)	0.88 (0.66-1.17)	0.37			0.92 (0.70-1.20)	0.52		
Smoking status (No vs Yes)	0.97 (0.74-1.27)	0.83			0.93 (0.73-1.20)	0.60		
BMI (≤22 vs >22)	0.97 (0.75-1.25)	0.80			0.95 (0.75-1.20)	0.65		
Tumor location (ref: Upper 1/3)						0.09		
Middle 1/3	0.80 (0.58-1.11)	0.18			0.85 (0.63-1.15)	0.30		
Lower 1/3	0.71 (0.53-0.96)	0.03			0.70 (0.53-0.92)	0.01		
Total	0.91 (0.44-1.87)	0.79			0.87 (0.44-1.72)	0.69		
Tumor size (≤5 vs >5)	1.57 (1.22-2.03)	0.0005	1.24 (0.95-1.60)	0.11	1.62 (1.28-2.06)	<0.0001	1.25 (0.98-1.59)	0.07
Pathologic T stage (ref: Upper 1/3)								
T2	2.85 (1.47-5.50)	0.002	1.97 (0.99-3.89)	0.05	2.94 (1.57-5.53)	0.001	2.02 (1.05-3.86)	0.03
T3	4.95 (2.69-9.09)	<0.0001	3.10 (1.65-5.84)	0.0005	5.56 (3.11-9.94)	<0.0001	3.52 (1.93-6.43)	<0.0001
Pathologic N stage (ref: N0)						<0.0001		
N1	1.77 (1.09-2.86)	0.02	1.46 (0.90-2.37)	0.13	1.76 (1.12-2.77)	0.01	1.47 (0.93-2.32)	0.10
N2	1.73 (1.09-2.76)	0.02	1.46 (0.91-2.34)	0.12	1.90 (1.23-2.92)	0.004	1.58 (1.02-2.44)	0.04
N3	4.21 (2.80-6.35)	<0.0001	3.31 (2.16-5.07)	<0.0001	4.29 (2.92-6.31)	<0.0001	3.33 (2.23-4.95)	<0.0001
Pathologic M stage (M0 vs M1)	2.50 (1.43-4.37)	0.001	1.27 (0.71-2.25)	0.42	2.34 (1.36-4.00)	0.002	1.19 (0.69-2.07)	0.54
Borrmann classification (ref: I)						0.001		
II	0.80 (0.46-1.39)	0.43			0.84 (0.50-1.41)	0.52		
III	1.45 (0.86-2.43)	0.16			1.50 (0.92-2.44)	0.10		
IV	1.37 (0.73-2.55)	0.32			1.37 (0.76-2.47)	0.30		

The univariate and multivariable Cox model regression was used for analyses. Abbreviations: BMI, body-mass index (calculated as weight in kilograms divided by height in meters squared); EIPL, extensive intraoperative peritoneal lavage; ref, reference.

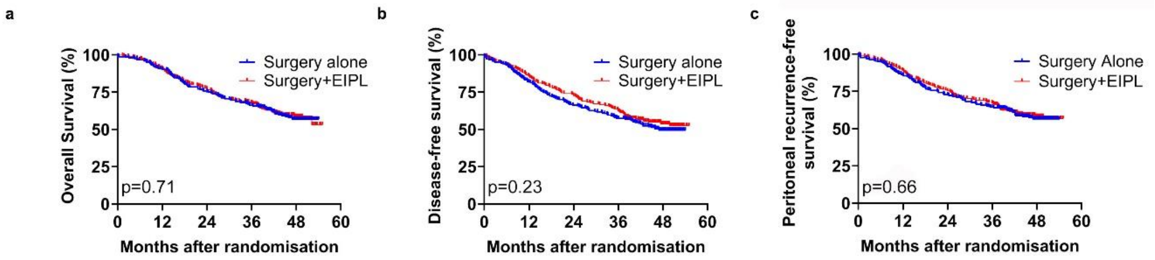

Number at risk (a)

Surgery alone	271	244	204	170	51	0
Surgery+EIPL	279	254	216	184	53	0

Number at risk (b)

Surgery alone	271	222	180	149	47	0
Surgery+EIPL	279	240	205	169	51	0

Number at risk (c)

Surgery alone	271	233	197	168	51	0
Surgery+EIPL	279	248	209	181	52	0

Supplementary Figure 1 Kaplan–Meier estimates of overall survival (a), disease-free survival (b) and peritoneal recurrence-free survival (c) in 550 patients with T3/4NxM0 disease by treatment group. Log-rank test was used tocompare both curves. EIPL, extensive intraoperative peritoneal lavage.

	3-year DFS rate (%) (95%CI)			Log-rank	Interaction
	Surgery alone	Surgery plus EIPL	HR (95%CI)	p value	p value
Age, years					0.79
<60	66.0 (58.2-73.7)	72.8 (65.6-80.0)	0.84 (0.57-1.24)	0.38	
>60	57.4 (50.2-64.5)	60.8 (53.7-67.8)	0.91 (0.67-1.22)	0.52	
Sex					0.85
Male	60.5 (54.3-66.6)	65.6 (59.5-71.7)	0.90 (0.68-1.18)	0.43	
Female	63.1 (52.8-73.4)	67.0 (57.8-76.2)	0.86 (0.54-1.36)	0.51	
Smoking status					0.48
No	62.1 (55.7-68.4)	64.3 (58.0-70.6)	0.94 (0.71-1.24)	0.65	
Yes	59.2 (49.7-68.7)	69.7 (61.0-78.3)	0.78 (0.51-1.18)	0.23	
BMI					0.61
<22	60.5 (53.1-67.9)	63.2 (55.7-70.8)	0.94 (0.67-1.30)	0.69	
>22	61.9 (54.4-69.4)	68.6 (61.7-75.4)	0.84 (0.60-1.17)	0.29	
Tumor location					0.20
Upper 1/3	59.6 (49.9-69.3)	56.7 (46.8-66.6)	1.00 (0.67-1.49)	0.99	
Middle 1/3	60.2 (50.0-70.5)	64.0 (53.8-74.1)	1.12 (0.71-1.75)	0.64	
Lower 1/3	63.6 (55.3-71.9)	74.3 (67.0-81.5)	0.68 (0.46-1.02)	0.06	
Total	54.5 (25.1-84.0)	60.0 (29.6-90.4)	0.79 (0.21-2.94)	0.72	
Tumor size, cm					0.69
<5	65.1 (58.8-71.5)	73.3 (67.4-79.3)	0.83 (0.61-1.13)	0.24	
>5	53.2 (43.8-62.6)	52.9 (44.0-61.9)	0.92 (0.64-1.31)	0.63	
Pathologic T stage					0.72
T1/2	91.5 (83.5-99.5)	97.7 (95.7-99.8)	0.69 (0.22-2.18)	0.53	
T3	71.6 (61.3-81.9)	73.7 (63.8-83.6)	0.82 (0.47-1.45)	0.50	
T4	50.5 (43.7-57.3)	56.8 (50.1-63.4)	0.89 (0.68-1.16)	0.37	
Pathologic N stage					0.92
N0	84.7 (76.4-93.0)	85.3 (77.3-93.3)	1.08 (0.54-2.16)	0.83	
N1	60.7 (48.4-72.9)	79.9 (70.6-89.3)	0.44 (0.24-0.81)	0.006	
N2	72.0 (62.2-81.7)	61.8 (50.9-72.8)	1.49 (0.89-2.48)	0.13	
N3	38.4 (29.4-47.4)	47.3 (38.1-56.6)	0.80 (0.58-1.12)	0.19	
Pathologic M stage					0.47
M0	62.3 (56.9-67.6)	67.2 (62.0-72.3)	0.89 (0.70-1.13)	0.33	
M1	22.2 (2.9-41.5)	30.0 (1.6-58.4)	0.56 (0.19-1.62)	0.28	
Borrmann classification					0.12
I	60.0 (38.5-81.5)	70.4 (53.1-87.6)	0.64 (0.25-1.61)	0.34	
II	67.3 (58.6-75.9)	75.5 (67.1-83.8)	0.64 (0.40-1.03)	0.07	
III	52.4 (44.3-60.5)	59.5 (51.9-67.0)	0.92 (0.67-1.25)	0.59	
IV	60.6 (43.9-77.3)	50.0 (30.8-69.2)	1.21 (0.58-2.51)	0.61	

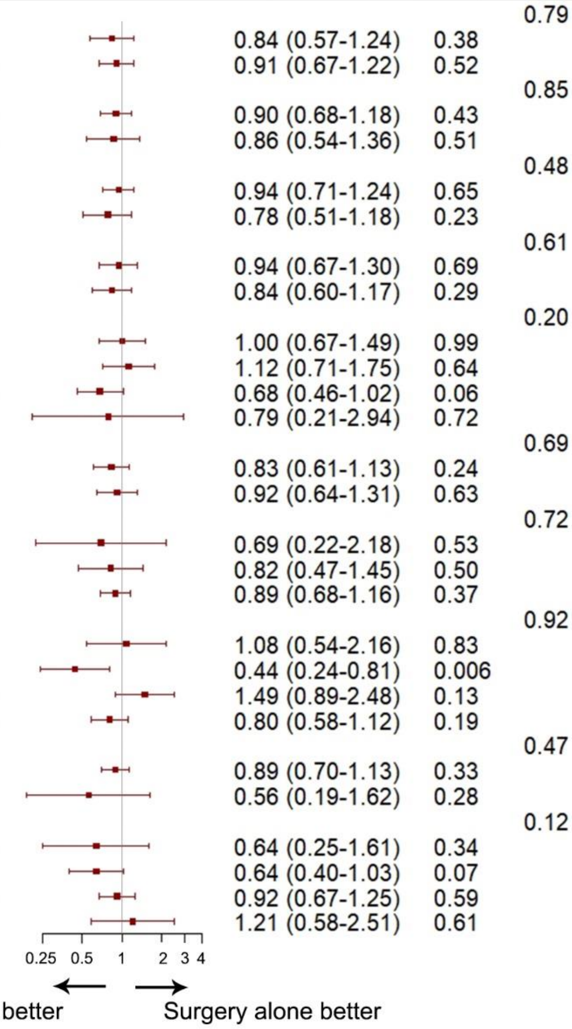

Supplementary Figure 2 Subgroup analyses of disease-free survival according to the clinicopathological characteristics of thepatients (n=662). Data are plotted as HR value (red squares) with the corresponding two-sided 95% CI (errorbars) based on the Cox regression method. EIPL, extensive intraoperative peritoneal lavage; DFS, disease-free survival; HR, hazard ratio; 95%CI, 95% confidence interval; BMI, body-mass index

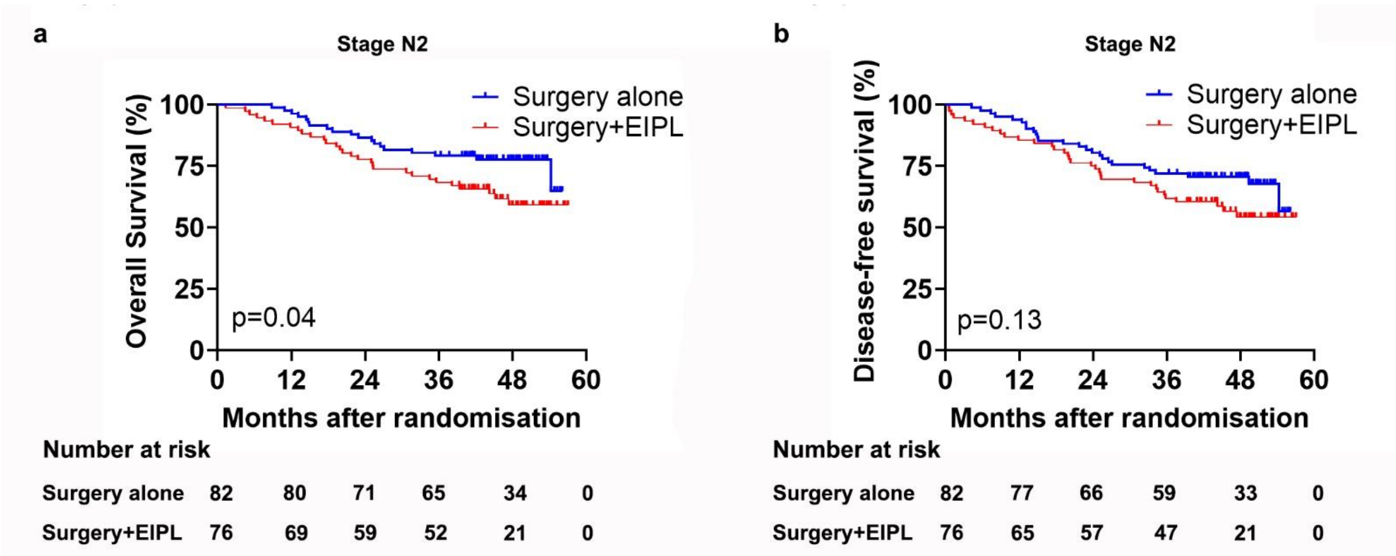

Supplementary Figure 3 Kaplan–Meier estimates of overall survival (a) and disease-free survival (b) in N2 patients bytreatment group. Log-rank test was used to compare both curves. EIPL, extensive intraoperative peritoneallavage.
