# Supplementary material for: Three-year outcomes of the randomized phase III SEIPLUS trial of extensive intraoperative peritoneal lavage for locally advanced gastric cancer
Source: Nat Commun. 2021 Nov 15;12:6598. doi: 10.1038/s41467-021-26778-8 (PMC8594430; doi:10.1038/s41467-021-26778-8)
Supplement: Supplementary file 3 — Reporting summary [file 41467_2021_26778_MOESM3_ESM.pdf]

nature research

Corresponding author(s): Dazhi Xu

Last updated by author(s): Aug 17, 2021

Reporting Summary

Nature Research wishes to improve the reproducibility of the work that we publish. This form provides structure for consistency and transparency in reporting. For further information on Nature Research policies, see our Editorial Policies and the Editorial Policy Checklist.

Statistics

For all statistical analyses, confirm that the following items are present in the figure legend, table legend, main text, or Methods section.

n/a	Confirmed	
☐	☒	The exact sample size (n) for each experimental group/condition, given as a discrete number and unit of measurement
☐	☒	A statement on whether measurements were taken from distinct samples or whether the same sample was measured repeatedly
☐	☒	The statistical test(s) used AND whether they are one- or two-sided<br>*Only common tests should be described solely by name; describe more complex techniques in the Methods section.*
☐	☒	A description of all covariates tested
☐	☒	A description of any assumptions or corrections, such as tests of normality and adjustment for multiple comparisons
☐	☒	A full description of the statistical parameters including central tendency (e.g. means) or other basic estimates (e.g. regression coefficient) AND variation (e.g. standard deviation) or associated estimates of uncertainty (e.g. confidence intervals)
☐	☒	For null hypothesis testing, the test statistic (e.g. F, t, r) with confidence intervals, effect sizes, degrees of freedom and P value noted<br>*Give P values as exact values whenever suitable.*
☐	☒	For Bayesian analysis, information on the choice of priors and Markov chain Monte Carlo settings
☐	☒	For hierarchical and complex designs, identification of the appropriate level for tests and full reporting of outcomes
☒	☐	Estimates of effect sizes (e.g. Cohen's d, Pearson's r), indicating how they were calculated

Our web collection on statistics for biologists contains articles on many of the points above.

Software and code

Policy information about availability of computer code

Data collection	Crabyter
Data analysis	SPSS (version: 17.0), R (version: 17.0), forestplot (version: NA)

For manuscripts utilizing custom algorithms or software that are central to the research but not yet described in published literature, software must be made available to editors and reviewers. We strongly encourage code deposition in a community repository (e.g. GitHub). See the Nature Research guidelines for submitting code & software for further information.

Data

Policy information about availability of data

All manuscripts must include a data availability statement. This statement should provide the following information, where applicable:

- Accession codes, unique identifiers, or web links for publicly available datasets
- A list of figures that have associated raw data
- A description of any restrictions on data availability

Raw data that support the findings of this study have also been deposited in Research Data Deposit public platform (http://www.researchdata.org.cn), with the Approval Number as RDDA2021002105.

Field-specific reporting

Life sciences study design

All studies must disclose on these points even when the disclosure is negative.

Sample size	This trial was designed to assess the superiority of surgery plus EIPL compared with surgery alone for the OS. We planned to detect an increase in 3-year OS benefit from 60% for the surgery alone group to 71% for the surgery plus EIPL group with 80% power at a two-sided 5% significant level, requiring 254 patients in each group.
Data exclusions	No data were excluded from these analyses.
Replication	NA
Randomization	Patients were assigned (1:1) to the surgery alone or EIPL plus surgery group with a block size of four. Randomization was stratified by each participating hospital. Allocation was executed by sealed opaque envelopes that contained the information of the random number and the procedure to which patients were assigned. The envelopes were opened after meticulous exploratory laparotomy.
Blinding	Patients were masked to treatment assignment. The investigators were not blinded to group allocation during data collection and analysis.

Reporting for specific materials, systems and methods

We require information from authors about some types of materials, experimental systems and methods used in many studies. Here, indicate whether each material, system or method listed is relevant to your study. If you are not sure if a list item applies to your research, read the appropriate section before selecting a response.

Materials & experimental systems

n/a	Involved in the study	
x		Antibodies
x		Eukaryotic cell lines
x		Palaeontology and archaeology
x		Animals and other organisms
	x	Human research participants
	x	Clinical data
x		Dual use research of concern

Methods

n/a	Involved in the study	
x		ChIP-seq
x		Flow cytometry
x		MRI-based neuroimaging

Human research participants

Policy information about studies involving human research participants

Population characteristics	We enrolled and randomly assigned 662 locally advanced gastric cancer patients: 329 patients to the surgery alone group (age:60.7± 10.7, male:245 (74.5%), female: 84 (25.5%))and 333 to the EIPL group(age:60.7± 10.6, male:233 (70.0%), female: 100 (30.0%)).
Recruitment	Surgeon from 11 hospitals in China used their existing patient population to determine which participants would meet the inclusion/exclusion criteria and then invited them to review the informed consent form. Participants at multiple centers were screened for each cohort to ensure enrollment was competitive and cohorts were balanced.
Ethics oversight	The complete names of the ethics committee and institutional review board are listed below:<br>1. Sun Yat-sen University Cancer Center<br>2. The First Affiliated Hospital of Anhui Medical University<br>3. The First Affiliated Hospital of University of Science and Technology of China<br>4. The First Affiliated Hospital of Wannan Medical College<br>5. Jiangxi Provincial Cancer Hospital<br>6. Anqing Municipal Hospital<br>7. Tianjin Medical University Cancer Institute and Hospital<br>8. The Second Affiliated Hospital of Zhejiang University School of Medicine<br>9. Yuebei People's Hospital<br>10. Jiangsu Cancer Hospital<br>11. Lishui Municipal Central Hospital

Note that full information on the approval of the study protocol must also be provided in the manuscript.

Clinical data

Policy information about clinical studies

All manuscripts should comply with the ICMJEguidelines for publication of clinical research and a completedCONSORT checklist must be included with all submissions.

Clinical trial registration	NCT02745509
Study protocol	ClinicalTrials.gov
Data collection	Data were collected by Crabyter (https://crabyter.sinyoo.net/). Between April 2016 and November 2017, we collected the baseline data from 11 centers in China. Between November 2017 and November 2020, we collected the follow-up data.
Outcomes	The primary endpoint was 3-year OS. The secondary endpoints included 3-year disease free survival (DFS), 3-year peritoneal recurrence-free survival and 30-day postoperative complication and mortality. OS is calculated from the date of randomization to the date of death whatever the cause or date of censoring. DFS was calculated from the day of randomization to the day of disease recurrence or death whatever the cause or date of censoring. Peritoneal recurrence-free survival was calculated from the day of randomization to the day of peritoneal recurrence or death irrespective of cause or date of censoring.
